# Involvement of claudin-7 in HIV infection of CD4(-) cells

**DOI:** 10.1186/1742-4690-2-79

**Published:** 2005-12-20

**Authors:** Junying Zheng, Yiming Xie, Richard Campbell, Jun Song, Samira Massachi, Miriam Razi, Robert Chiu, James Berenson, Otto O Yang, Irvin SY Chen, Shen Pang

**Affiliations:** 1 UCLA School of Dentistry, UCLA Dental Institute, and Jonsson Comprehensive Cancer Center, 10833 Le Conte Ave., Los Angeles, CA 90095, USA; 2 Departments of Medicine and Microbiology & Immunology, and UCLA AIDS Institute, David Geffen School of Medicine at UCLA, 10833 Le Conte Ave., Los Angeles, CA 90095, USA; 3 Department of Medicine, Div. of Infectious Diseases, David Geffen School of Medicine at UCLA, 10833 Le Conte Ave., Los Angeles, CA 90095, USA; 4 Institute for Myeloma & Bone Cancer Research, 9201 Sunset Blvd., Suite 300, West Hollywood, CA90069, USA

## Abstract

**Background:**

Human immunodeficiency virus (HIV) infection of CD4(-) cells has been demonstrated, and this may be an important mechanism for HIV transmission.

**Results:**

We demonstrated that a membrane protein, claudin-7 (CLDN-7), is involved in HIV infection of CD4(-) cells. A significant increase in HIV susceptibility (2- to 100-fold) was demonstrated when CLDN-7 was transfected into a CD4(-) cell line, 293T. In addition, antibodies against CLDN-7 significantly decreased HIV infection of CD4(-) cells. Furthermore, HIV virions expressing CLDN-7 on their envelopes had a much higher infectivity for 293T CD4(-) cells than the parental HIV with no CLDN-7. RT-PCR results demonstrated that CLDN-7 is expressed in both macrophages and stimulated peripheral blood leukocytes, suggesting that most HIV virions generated in infected individuals have CLDN-7 on their envelopes. We also found that CLDN-7 is highly expressed in urogenital and gastrointestinal tissues.

**Conclusion:**

Together these results suggest that CLDN-7 may play an important role in HIV infection of CD4(-) cells.

## Background

Human immunodeficiency virus (HIV) transmission through sexual intercourse accounts for the majority of infections. It must cross the epithelium during transmission, because the primary targets for HIV infection, CD4(+) cells, are protected by epithelial lining. However, the mechanism by which the virus transverses the epithelia covering the reproductive tract, the oral cavity, the gastrointestinal tract, or other tissues during viral transmission is poorly understood. This is an important question to investigate, because the epithelium, which is composed of stratified CD4(-) epithelial cells, protects the interface between host and environment (e.g., urogenital, gastrointestinal tract) or between organs and fluid spaces (prostate, kidney).

HIV may not utilize the mechanism of binding between gp120 on virions and CD4 molecules to infect epithelial cells, because these cells are CD4(-). One possible mechanism is that HIV utilizes lesions in the mucosal surface to invade underlying lymphoid cells [1,2]. Conversely, it has been shown that lesions need not be present for the virus to cross the epithelial barrier [3-5]. Therefore, it is likely that HIV can penetrate epithelial layers by other mechanism(s). HIV may enter epithelial cells by a simple in-and-out means [6] or by transcytosis [7], whereby the cells passing across are not infected. However, recent reports demonstrate that many types of epithelial cells can be infected with HIV-1 [8-12], and that viral replication also occurs in infected epithelial cells.

Two possible mechanisms by which HIV infects CD4(-) cells have been proposed. Some reports suggest that the HIV gp120 surface glycoprotein binds to galactosylceramide (GalCer) [13-15] or chemokine receptors, including CXCR4 and CCR5, on the surface of CD4(-) cells [15-19], and that this binding initiates HIV entry into CD4(-) cells. Therefore, gp120 would be a key protein for HIV infection of CD4(-) cells. However, our previous results demonstrated that HIV infects many types of CD4(-) cells, some without surface gp120 [20-22]. Therefore, CD4(-) cell infection can be gp120-independent; i.e., the presence of gp120 glycoprotein molecules on the viral surface is not crucial for CD4(-) cell infection.

An important approach to understanding gp120-independent HIV infection is to identify the elements involved in this mechanism of infection. To do so, we compared a CD4(-) cell line that is highly susceptible to HIV infection to another cell line that has low susceptibility. By screening membrane proteins that are specifically expressed in the cell line highly susceptible to HIV, it is possible to identify the genes that are involved in HIV infection.

Our previous studies demonstrated that HIV efficiently infects the prostate cancer cell line, LNCaP [22]. When a viral load of approximately 100 ng p24 was used for infection of 10^4 ^cells in culture, approximately 3–20% of LNCaP cancer cells were infected. The concentration of 100 ng p24/0.5 ml is similar to the viral load found in patients in the acute phase of infection. Infection of LNCaP cells is gp120-independent, because HIV with or without gp120 on its envelope is equally infectious for these cells, and antibodies against gp120 do not block infection. It is expected that certain proteins specifically expressed on the surface of this cell line are responsible for gp120-independent HIV infection.

We used subtractive cDNA cloning to identify a gene specifically expressed in LNCaP cells but not in the 293T and HeLa cell lines, which are not susceptible to HIV infection [20]. Here we characterize the role of this protein, claudin-7 (CLDN-7), in gp120-independent HIV infection.

As previously described [20], we generated Env(-) HIV_NL4-3 _by deleting a fragment of 581 bp from the *env *coding region. This deletion truncates the gp120 envelope protein and introduces a frameshift into downstream gp41, thereby abrogating gp120 and gp41. The modified HIV also contains a reporter gene, the enhanced green fluorescent protein (EGFP). Insertion of the EGFP gene enables direct and sensitive detection of HIV infection. Previous reports have demonstrated that the substitution of the *nef *gene with EGFP does not alter viral infectivity [23-25]. To examine gp120-independent infection, gp120 and gp41 were deleted from the HIV_NL4-3 _genome, which eliminates the interference of viral envelope proteins. We have successfully utilized this modified viral strain to study gp120-independent infection, and therefore used this strain for the studies described herein [20,22].

Our previous studies demonstrated that a membrane protein, claudin-7 (CLDN-7), is expressed in the HIV-susceptible cell line, LNCaP, but not in the HIV non-susceptible cell line, 293T [26]. We studied the relationship of the expression of this protein and infection by HIV. In the described study, we transfected 293T cells with cloned CLDN-7, then characterized the infection of these cells with EGFP-modified HIV_NL4-3_.

## Results

### The prostate cell line, LNCaP, is highly susceptible to gp120-independent HIV-1 infection

**Figure 1:**
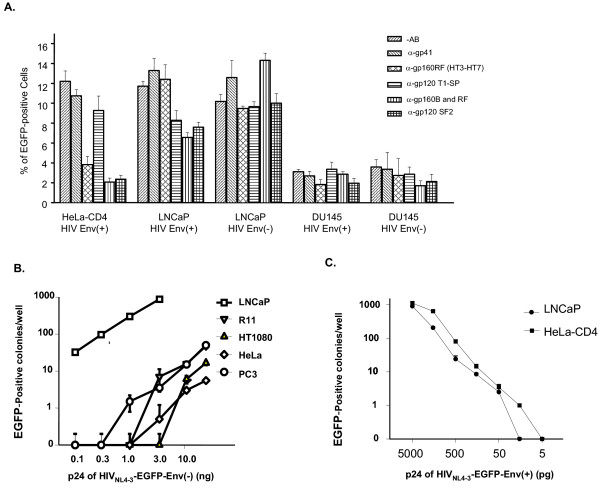
HIV infection of LNCaP cells. LNCaP or HeLa-CD4 cells in 24-well culture plates (10^4 ^cells/well) were infected with HIV either with or without gp120 protein on its envelope [Env(-) and Env(+) HIV]. A) A significantly high percentage of EGFP-positive cells was demonstrated in the LNCaP cell cultures infected by HIV Env(-) virus (9–14%). Infection of HeLa-CD4 cells by HIV Env(+) was used as a positive control to assess anti-gp120 or -gp160 function of the antibodies. Infection of HeLa cells was performed as a negative control. The infections of HIV either with or without Env showed very low infectivity for HeLa cells, as demonstrated with no EGFP-positive cells in the infected culture, or occasionally there were one or two EGFP-positive colonies. In other experiments, we also infected HeLa-CD4 cells with Env(-) HIV_NL4-3_, and found that the Env(-) HIV strain did not infect HeLa-CD4. These results have been previously reported (22). B) Infection of CD4(-) cell lines by HIV_NL4-3_-Env(-)-EGFP virus prepared from an oral epithelial cell line derived from a patient. The cell line was established and maintained in our laboratory. C) Infection of the LNCaP and HeLa-CD4 cell lines by HIV at various concentrations. Because the figure is in log scale, the standard deviations do not appear clearly. These are: 1) LNCaP: 900 ± 38 (5 ng), 205 ± 11(1.5 ng), 24 ± 5.6 (0.5 ng), 8.5 ± 0.7 (0.15 ng), 2.5 ± 0.7 (50 pg); and 2) HeLa-CD4: 1114 ± 115 (5 ng), 638 ± 47 (1.5 ng), 80 ± 10 (0.5 ng), 14.5 ± 2.1 (0.15 ng), 3.5 ± 0.71 (50 pg), 1 ± 0 (15 pg).

We previously reported that by employing a gp120-independent infection mechanism, HIV-1 virus infected several CD4(-) cell lines, including oral cell lines Tu139 and Tu177, prostate cell lines LNCaP and DU145, and the fibroblast cell line, HT-1080. However, the infection levels of 293T cells by HIV are low [20]. The CD4(-) cell line, LNCaP, demonstrated the highest HIV susceptibility [22]. When 10^4 ^LNCaP cells per well in a 24-well plate were infected with virus at a concentration of 100 ng/ml p24, more than 10% of the cells were infected by virus prepared from either 293T cells (Figure 1A) or an oral epithelial cell line (Figure 1B). Because there is a partial gp120 sequence remaining (279 of 509 amino acid residues), it was necessary to ascertain whether the truncated gp120 has any effects upon infection of LNCaP cells. Antiserum against gp120 or gp160, the precursor of gp120 and gp41, was used to block the interaction between the truncated gp120 and its potential ligands. Infection of LNCaP cells with HIV-1 Env(-) virus was not significantly affected, suggesting that HIV infection of LNCaP cells is gp120-independent. It should also be noted that the infection rate of LNCaP cells by HIV was similar to that of HeLa-CD4, suggesting that the infectivity of HIV-1 for some types of CD4(-) cells is high. Because 12% HIV infectivity of HeLa-CD4 occurs through the binding of gp120 to CD4 molecules on the cell surface of HeLa-CD4 cells, we would expect that infection of LNCaP by HIV Env(-) virus would occur through binding of unknown proteins of the virus and the cells, and the binding affinity should be comparable to that between gp120 and CD4 molecules.

To accurately compare the infectivity for LNCaP and HeLa-CD4 cells by HIV_NL4-3 _containing the EGFP gene, we infected these two cell lines with different concentrations of virus (Figure 1C). When LNCaP cells in 24-well plates were infected with 50 pg of p24 counts of virus, less than five infected colonies per well were detected. When the cells were infected with virus containing 15 pg of p24, no infected colonies were detected. When HeLa-CD4 cells were infected with virus at similar or lower concentrations, we found that they could be infected by virus containing 15 pg of p24. However, when the concentration of virus was diluted to 5 pg of p24, no positive colonies were detected in the infected cell cultures, suggesting that the sensitivity of LNCaP cells for HIV is approximately 3-fold lower compared with that of HeLa-CD4 cells (Figure 1C).

To ensure that infection of LNCaP cells by virus generated from 293T cells is not caused by potential contamination of viruses that can modify the HIV envelope (e.g., amphotropic murine viruses), we tested 293T cells derived from various sources. The results were similar to those shown in Figure 1A, with much higher infectivity for LNCaP cells as compared to other cell lines, including 293T, HeLa, and DU145. It is unlikely that all of the 293T cells from different sources were contaminated; in other words, it is unlikely that the virus generated from 293T cells is modified by any contaminated amphotropic viruses.

### CLDN-7^211 ^or CLDN-7^158 ^increases susceptibility of CD4(-) cells to HIV infection

**Figure 2:**
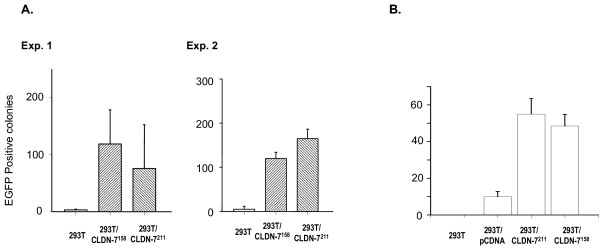
Effect of CLDN-7 molecules upon infection of 293T cells by HIV_NL4-3_. A) HIV_NL4-3 _Env(-) virus infection of either CLDN-7^211^- or CLDN-7^158^-modified 293T cells. In experiment 1, HIV Env(-) at 50 ng of p24 was used to infect 10^4 ^cells in each well; in experiment 2, 10^4 ^cells were infected by virus at 100 ng of p24. B) HIV_NL4-3 _Env(+) virus infection of either CLDN-7^211^- or CLDN-7^158^-modified 293T cells. Plasmid pCDNA3-transfected 293T cells were also used as a control.

Using subtractive hybridization, we identified the CLDN-7 gene, which is highly expressed in prostate cells but not in 293T cells [26]. Two transcripts of this gene have been identified, one encoding a peptide of 211 amino acid residues, and the other, 158 [26]. The plasmid vectors that carry CLDN-7^211 ^or CLDN-7^158 ^were used to transfect 293T cells. Two days post-transfection, the cells were plated into 24-well plates for viral infection. The susceptibility of these transfected cells to Env(-) HIV_NL4-3 _infection is significantly increased. Compared with non-transfected cells, the numbers of infected cells in the transfected 293T cell cultures were over 10-fold higher (Figure 2A). These results suggest that the presence of either CLDN-7^211 ^or CLDN-7^158 ^on the surface of CD4(-) cells increases their susceptibility to HIV-1 infection via a gp120-independent mechanism. In a separate experiment, we also tested CLDN-7-transfected 293T cells using EGFP-modified Env(+) HIV_NL4-3_. The results were very similar to those using Env(-) HIV_NL4-3 _(Fig. 2B).

### Antibodies specific to CLDN-7 block gp120-independent infection

**Figure 3:**
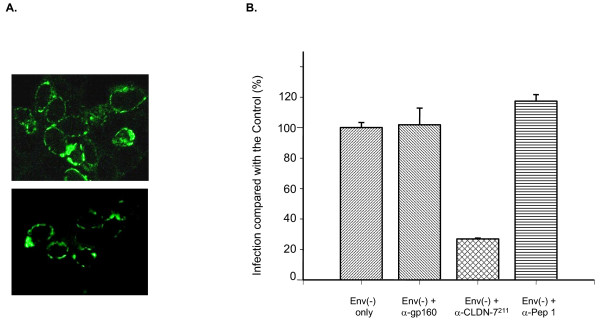
Inhibition of HIV infection by antibodies specific to CLDN-7. A) Immunostaining of CLDN-7^211 ^(upper panel) and CLDN-7^158 ^(lower panel) by CLDN-7-specific polyclonal antibodies. 293T cells (5 × 10^4^) were plated into 35-mm plates 24 hours prior to transfection. CLDN-7 plasmids (3 μg) were used for transfection of each 35-mm plate. At two days post-transfection, the cells were immunostained. CLDN-7 antibodies were added into the plates overnight at 4°C. B) Antisera from NIH (anti-gp160, cat. 191), Zymed (anti-CLDN-7, 0.25 mg/ml), or made by us were added to LNCaP cell cultures at 1:100 v/v 10 minutes prior to adding HIV. Six days post-infection, EGFP-positive cells were counted. Infection of LNCaP cultures with no antibodies was set as the control. EGFP-positive cells in the culture treated with antibodies against CLDN-7 demonstrated 27% ± 0.6% viral infection compared to the control with no antibodies in the cell culture.

Immunostaining demonstrated that CLDN-7 is a membrane protein, similar to other claudins (Figure 3A). To determine whether CLDN-7 is the key protein involved in gp120-independent infection, we used antibodies specific to CLDN-7 to block HIV infection. Polyclonal antibodies against either CLDN-7 (Zymed Laboratories) or gp160 (cat. #191, NIH AIDS Reagent Program) were added into LNCaP cell cultures. It was expected that the extracellular domains of CLDN-7 on the surface of LNCaP cells could be bound by CLDN-7 antibodies, and the binding of these antibodies to CLDN-7 would disrupt the binding of HIV to CLDN-7. As a result, HIV infection of CD4(-) cells should be decreased. Our results demonstrated that the antibodies for CLDN-7 decreased HIV infectivity for LNCaP cells (Figure 3B), whereas gp160-specific antibodies did not show inhibition, suggesting that CLDN-7 on the surface of LNCaP cells is involved in HIV infection. Because we used the gp120-negative virus strain for infection and antibodies against gp160 do not show inhibition, we believe that infection of LNCaP cells occurs via a gp120-independent mechanism.

We also tested a preparation of polyclonal antibodies we generated against a peptide with the sequence CVTQSTGMMSCKMYD. The peptide sequence corresponds to aa positions 44 to 58 of CLDN-7. A cell-labeling assay demonstrated that this antibody preparation is able to bind CLDN-7 on the cell membrane (data not shown). However, it did not significantly block HIV infection of LNCaP cells, suggesting that this sequence region in CLDN-7 may not be essential for HIV infection (Figure 3B).

### Increased infectivity for viruses generated with CLDN-7^211 ^or CLDN-7^158 ^on their envelopes

**Figure 4:**
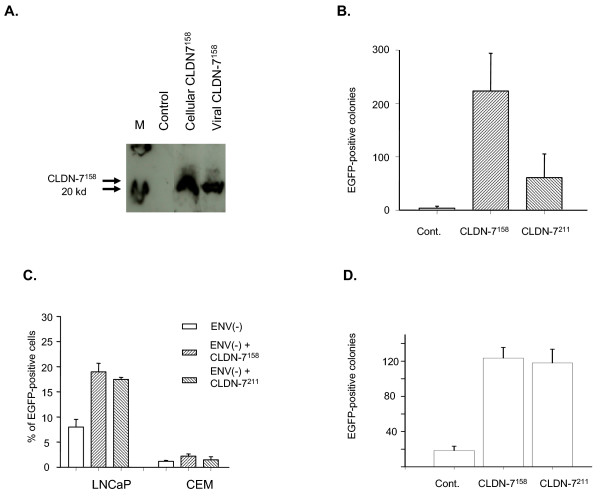
Infectivity of CLDN-7^211^- or CLDN-7^158^-modified Env(-) virus. A) Western blot of proteins isolated from the virus generated from the transfected 293T cells by pNL4-3-EGFP-Env(-) and CLDN-7. Cell culture medium collected from cells transfected with CLDN-7 was used as a control to assess the background of CLDN-7 in microvesicles. Cellular proteins isolated from CLDN-7 plasmid-transfected cells were used as a positive control. The monomers of CLDN-7 are approximately 22 kd. Lane M is a molecular marker lane. The monomers of CLDN-7 are approximately 22 kd. B) CLDN-7-modified HIV_NL4-3 _Env(-) virus showed significantly higher infectivity for 293T cells. C) Infection of either LNCaP or CEM CD4(+) T-lymphocyte cell lines by CLDN-7-modified HIV, with approximately a 1.5- to 2-fold increase of viral infection. D) CLDN-7-modified HIV_JRCSF _virus with intact gp120 showed significantly higher infectivity for a CD4(-) cell line, PC-3, than the virus with no CLDN-7 on its surface.

A possible explanation for increased HIV-1 infectivity for the CLDN-7-transfected cells is that gp120-independent HIV-1 infection is mediated by an interaction between a cellular protein on the viral envelope and CLDN-7 expression on the surface of transfected 293T cells. We hypothesized that the ligands for CLDN-7 are present on the surface of many types of cells. If HIV has CLDN-7 on the surface of its envelope, it is expected that CLDN-7 on the viral surface can bind to the ligands for CLDN-7 on target cells, so that HIV infection can be increased. To confirm this, we prepared HIV expressing CLDN-7 on the viral surface by co-transfecting a plasmid containing CLDN-7 (either CLDN-7^211 ^or CLDN-7^158^) with a plasmid that contains the HIV genome into 293T cells. Because HIV uses a patch of the host cell membrane as its envelope, it was expected that the CLDN-7 molecules on the membranes of CLDN-7 gene-transfected 293T cells could be taken up by HIV virions during viral assembly. Western blotting confirmed this to be the case (Figure 4A), and CLDN-7 was detected. We also collected the medium from 293T cell cultures transfected by the CLDN-7 gene as a negative control to assess the contribution of CLDN-7 in membranous particles, termed microvesicles, because viral preparations are generally contaminated with these particles [27]. As shown in Figure 4A, no CLDN-7 protein was detected in the CLDN-7-transfected 293T culture medium, suggesting that either the amounts of microvesicles from transfected 293T were very low or the microvesicles generated from transfected 293T cells do not contain significant amounts of CLDN-7 protein. Therefore, the contribution of CLDN-7 derived from microvesicles in viral preparations is insignificant. We used the CLDN-7-modified Env(-) EGFP-HIV_NL4-3 _to infect 293T, LNCaP, and CEM cells. CLDN-7^158^-modified HIV_NL4-3_-EGFP-Env(-) demonstrated approximately 100-fold higher activity when infecting 293T cells (Figure 4B) compared with the parental HIV that does not have CLDN-7. The infection efficiencies in LNCaP and CEM cells by CLDN-7-modified viruses also showed a significant increase. These results suggest that CLDN-7 expressed on either the surface of the target cells or on the surface of the HIV envelope increases gp120-independent infection.

We also used EGFP-modified HIV_JRCSF_, a patient-derived R5 strain, to infect CD4(-) cells. Using co-transfection, we generated CLDN-7-modified HIV_JRCSF _from 293T cells. Because both CLDN-7-negative and -positive HIV_JRCSF _did not infect 293T cells, we infected another CD4(-) cell line, PC-3. The results demonstrated that CLDN-7 also significantly increases HIV_JRCSF _infectivity for this CD4(-) cell line.

### Expression of CLDN-7 mRNA in peripheral blood lymphocytes and macrophages

**Figure 5:**
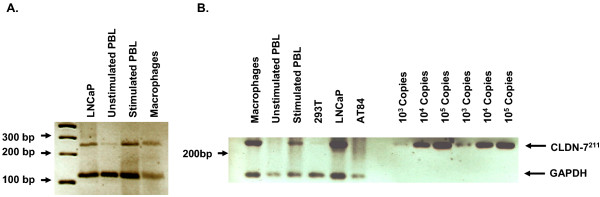
Expression of CLND-7^211 ^in PBL and macrophages. RNA isolated from unstimulated PBL, interleukin 2-stimulated PBL, macrophages, and control cell lines was analyzed using RT-PCR. RNA samples were reverse transcribed using the oligo-dT primer, followed by PCR using the primers 5'-CTCCTCTGACTTCAACAGCG-3' and 5'-TGTTGCTGTAGCCAAATTCG-3' to detect the glyceraldehydes-3-phosphate dehydrogenase (GAPDH) gene as RNA standard, and the primers described previously [26] for detecting CLDN-7 RNA. Panels A and B are RT-PCR from different samples.

Because CLDN-7 on the cell membrane significantly increased susceptibility of CD4(-) cells to HIV-1, it was expected that virus generated from cells that express CLDN-7 would have higher infectivity for CD4(-) cells than virus generated from cells that do not. It is important to quantify the expression profile of the CLDN-7 gene in CD4(+) cells, including T-cells and macrophages. We used RT-PCR to examine the expression of CLDN-7 in peripheral blood lymphocytes (PBL) and macrophages, and found that both stimulated PBL and macrophages express CLDN-7 (Figure 5). Expression of CLDN-7 in PBL and macrophages suggests that HIV produced from these two types of cells carries CLDN-7 on its envelope. Because stimulated CD4(+) T-lymphocytes in PBL and macrophages are the major types of cells hosting and generating HIV in patients, virus derived from patients should also infect certain types of CD4(-) cells during HIV transmission.

### Expression of CLDN-7 in other tissues

**Figure 6:**
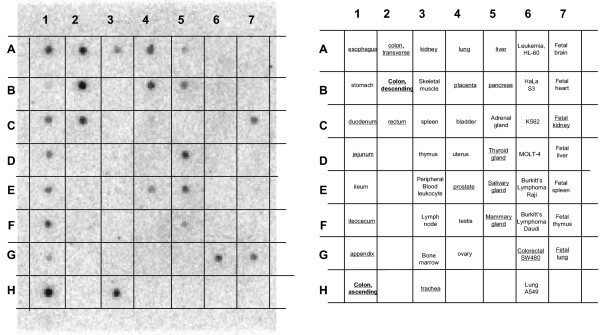
Expression of CLDN-7 molecules in human tissues. A nylon filter preloaded with RNA from various tissues from BD Clontech (Palo Alto, CA) was used to assess the expression levels of CLDN-7 in various tissues. The left panel shows the hybridization of the tissue samples in the filter by a CLDN-7 probe containing the coding region, and the right panel shows the corresponding tissues.

We used the coding region of CLDN-7^211 ^as a probe to hybridize mRNAs derived from more than 50 different tissue types and cell lines, and found that CLDN-7 is expressed in certain tissues in the urogenital and gastrointestinal systems, such as the colon, intestine, trachea, kidney, lung, and prostate (Figure 6). Infection of epithelial cells in these tissues and organs by HIV-1 has been reported, and they are the sites of many AIDS-related symptoms [10,14,28-33]. Thus, studies of this gene and its relationship with gp120-independent HIV infection will be important for understanding HIV-1-related pathologic effects.

## Discussion

Results from our previous studies and others have revealed that CD4(-) cells can be infected by HIV, so it is important to understand this process in the context of viral transmission, whereby HIV transverses CD4(-) epithelial cell layers and infects CD4(+) T-lymphocytes and macrophages. In addition, infected CD4(-) cells, such as cells in the central nervous system, may serve as viral reservoirs. Some reports demonstrated that the binding of gp120 to GalCer or chemokine receptors is the mechanism of CD4(-) cell infection; however, only particular types of HIV were reported to infect certain types of CD4(-) cells [13-19]. Our previous studies demonstrated that both the X4 and R5 types of HIV can infect CD4(-) cells [20-22], and for many types of CD4(-) cells, gp120 is not required for infection.

Our results demonstrated that Env(-) HIV is still able to infect many types of cells via a gp120-independent mechanism. Our results demonstrated that although Env(-) HIV could not efficiently infect CD4(+) cells, it has similar infectivity for CD4(-)cells. It is possible that in some infected cells, HIV can down-regulate the expression of Env proteins to evade the immune response. If that were the case, there should be a high percentage of Env(-) HIV present in patients, which may be able to infect some types of CD4(-) cells, such as neurons and glial cells. The infection of brain cells may be a major hindrance of viral eradication because they have a long life-span,.

**Figure 7:**
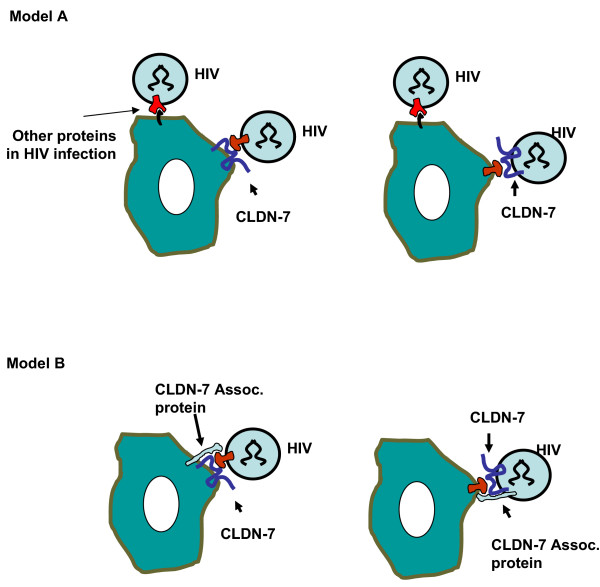
Two models for gp120-independent HIV infection. Model 1: HIV may use cellular proteins that are anchored to its envelope to bind to either CLDN-7 or other proteins. In LNCaP, both CLDN-7 and associated proteins involved in gp120-independent infection are present. In 293T cells, neither CLDN-7 nor the other infection-related membrane protein(s) is present. Expression of CLDN-7 in 293T may increase infectivity, but the levels of infection of the CLDN-7-modified 293T may still be significantly lower than those for LNCaP. Model 2: HIV may use cellular proteins that are anchored to its envelope to bind to a CLDN-7-associated complex. Although transfection of CLDN-7 can express this protein on the cell surface, the lack of the CLDN-7-associated protein decreases the binding of virus to the target cells.

It is important to identify the genes involved in gp120-independent infection. As described here, we found that a membrane protein, CLDN-7, can serve as a receptor for HIV-1 infection of CD4(-) cells or as a ligand on the viral envelope. CLDN-7 belongs to the claudin membrane protein family. Some claudins, such as CLDN-1 and CLDN-2, are involved in formation of tight junctions (TJ) between cells [34,35], while others may serve as receptors. As previously described, human claudin4 (CPE-R) is a receptor for the clostridium perfringens enterotoxin [36]. It is possible that CLDN-7 plays a role as the receptor for a protein ligand that is expressed on the surface of HIV viral particles. Our results have also demonstrated that CLDN-7 can be taken up as a component of viral particles. HIV may also use this protein to bind to target cells for infection. Based on our results and general HIV biology, we propose the model shown in Figure 7. In this model, the interaction of CLDN-7 with its ligand helps the virus to bind to CD4(-) cells; however, there may be other proteins that can also do this. Although expression of CLDN-7 in 293T cells significantly increased HIV infection, infection of CLDN-7-expressing 293T cells was still significantly lower than HIV infection of LNCaP cells. We therefore believe that CLDN-7 is not the only protein involved in HIV infection of CD4(-) cells. It is possible that the association of CLDN-7 with another protein can cause more efficient infection. In LNCaP cells, both CLDN-7 and its associated protein are expressed. In 293T or HeLa cells, expression levels of both CLDN-7 and its associated protein may be very low. Although we can use transfection to express CLDN-7 in 293T cells, the expression levels of the CLDN-7-associated protein may not be correspondingly increased. Therefore, addition of CLDN-7 to 293T cells can only partially increase levels of HIV infectivity, approximately 10% of that of LNCaP cells.

Because many tissues of the gastrointestinal and urogenital systems express the CLDN-7 gene, the cells in these tissues may be more susceptible to HIV-1 infection. These results are consistent with clinical data, with HIV-1 infection of epithelial cells of the oral mucosa, colon, intestine, and kidney being reported in patients [10,14,28-33]. In addition, because the virus uses a patch of cellular membrane as its envelope, when the virus is generated from cells in which the CLDN-7 protein is expressed, the virus also expresses this protein on its envelope. The presence of CLDN-7 molecules on the viral envelope may greatly increase its capacity for infecting other CD4(-) cells. It is expected that the viruses generated from infected PBL, macrophages, colon, intestine, trachea, kidney, lung, and prostate express this protein on their envelopes. This portion of the virus may have greater infectivity for CD4(-) cells compared to HIV virions that do not have CLDN-7 on their envelopes.

Our previous studies demonstrated that HIV can also use a gp120-independent mechanism by which to infect CD4(+) cells [22]. Because macrophages express much lower levels of CD4 molecules on the cell surface, it is expected that expression of CLDN-7 on the surface of macrophages may help HIV to infect these cells.

## Conclusion

Our results demonstrate that the presence of CLDN-7 on the surface of target cells increases viral susceptibility. Because CLDN-7 is expressed in organs related to HIV transmission and HIV pathogenicity (including the colon, kidney, lung, uterus, and oral tissue), it is expected that this protein is associated with HIV infection of CD4(-) cells in these organs, and is related to viral transmission or pathogenicity. Our results also demonstrated that virus generated from CLDN-7-transfected 293T cells has two- to 100-fold higher levels of infectivity, suggesting that the presence of CLDN-7 or other types of cellular membrane proteins on the viral envelope is important for viral infection. Because CLDN-7 is expressed in activated PBL and macrophages, and these two types of cells serve as HIV hosts, it is very likely that most HIV particles carry CLDN-7 on their surface. Therefore, it is very likely that this protein plays important roles in HIV infection of CD4(-) cells in humans.

## Materials and methods

### Cell culture

Cell lines LNCaP, DU145, HT1080, R11, HeLa, CEM, and 293T were purchased from American Type Culture Collection (ATCC) or from other laboratories, as described [20,22]. Cell lines LNCaP, PC-3, and DU145 are derived from the prostate, 293T from the embryonic kidney, CEM is a CD4(+) T-lymphocyte cell line, R11 is a renal carcinoma cell line, HT1080 is a fibroblast cell line, and HeLa is a from cervical cancer cell line. We also prepared HIV from an oral epithelial cell line derived from a patient. All cell lines were maintained in RPMI medium supplemented with 10% fetal bovine serum (FBS).

### The CLDN-7 gene

Using a subtractive hybridization method combined with RT-PCR, followed by screening prostate cDNA libraries, we obtained three full-length cDNA clones. Sequence analysis demonstrated that these cloned cDNA sequences are homologous to human CLDN-7. Two of these three cDNA clones encode a peptide of 211 amino acid residues identical to that in Genbank. The third encodes a peptide of 158 amino acid residues, which is a truncated form of CLDN-7 lacking 53 amino acid residues at the C-terminus [26]. Previous studies have demonstrated that both the full-length (CLDN-7^211^) and the truncated (CLDN-7^158^) forms of CLDN-7 are highly expressed in LNCaP but not 293T cells, and are expressed at low levels in HeLa cells [26]. Both isoforms of CLDN-7 were inserted into a plasmid vector downstream of the CMV promoter.

### Viral preparation and titration

To obtain HIV-1 Env(-) virus, we transfected either the 293T cell line or an oral epithelial cell line established in our laboratory with plasmid pNL4-3-EGFP-Env(-), which contains a modified HIV_NL4-3 _viral genome [20]. The modified HIV-1_NL4-3 _genome has deletions in *env *(581 bp) and *nef *(222 bp), and insertion of the EGFP gene, as previous described [20]. At 16 hours post-transfection, medium containing the plasmid was removed from transfected cell cultures. The transfected cell cultures were then washed with serum-free medium before adding new culture medium supplemented with 10% FBS. The medium containing virus was collected at days 2, 3, and 4 post-transfection. To remove cell debris, all the viral preparations were passed through a 0.2-micron filter. The collected viral stocks were titrated by p24 assays. The human 293T cell line does not express CLDN-7. Therefore, virus generated from this cell line is CLDN-7-negative. CLDN-7-positive virus was generated by co-transfection of 293T cells with both pCDNA3.1-CLDN-7 (CLDN-7^211 ^or CLDN-7^158^) and the modified pNL4-3-EGFP-Env(-). Viruses generated from these co-transfections carried the CLDN-7 protein on their envelopes and were also titrated by p24 assays.

EGFP gene-modified HIV_JRCSF_, a patient-derived R5 strain, was constructed using a similar approach. A part of the *nef *gene was substituted by the EGFP gene. We generated infectious HIV_JRCSF _by transfection of 293T cells. This virus can infect cells that express both CCR5 and CD4 on the cell surface, and replicates in infected cells. Because it contains a viral genome that is almost intact but lacks the *nef *gene, this virus alone propagates in CCR5(+)/CD4(+) cells.

### Infection of cell cultures

Cells (5 × 10^3^) were plated into each well of 24-well plates 24 hours prior to infection. During infection, a viral aliquot with a p24 count of 100 ng was added into each well of cell cultures. The final volumes in each well were adjusted to 0.5 ml so that the concentration of virus was 100 ng of p24 counts of virus in 0.5 ml of medium. At 16 hours post-infection, the cell cultures were washed.

### DNA transfection

Liposome FUGENE-6 (Roche Molecular Biochemicals, Indianapolis, IN) was used to transfect the CLDN-7 plasmid into 293T cells. Cells (2 × 10^4^) were plated into each well of 24-well plates 16 hours prior to lipofection. Plasmid DNA (2.0 μg) was mixed with FUGENE-6 liposome in 50 μl of RPMI medium for 10 minutes at room temperature before addition to cell cultures. At 8 hours [post-transfection?], the cell cultures were washed once and fresh medium then added.

### Determination of EGFP expression

The expression levels of EGFP were determined by counting EGFP- positive cells by fluorescent microscopy or by fluorescent-activated cell sorting (FACS).

### Western blotting

Cell culture medium from transfected 293T cells with pCDNA3.1-CLDN-7 and pNL4-3-EGFP-Env(-) or only pCDNA3.1-CLDN-7 was collected two to four days post-transfection. The collected medium was ultracentrifuged at 16,000 rpm for one hour at 4°C. The pellets were resuspended with 50 μl of protein lysis buffer (0.5% NP40, 1.0% glycerol, 0.1% β-mercaptoethanol, 40 mM Tris, pH6.8). The viral lysates were incubated at 37°C for 5 minutes before SDS-polyacrylamide gel electrophoresis (PAGE). Protein concentrations were determined by a standard protein assay (BioRad, Hercules, CA). Aliquots representing 2.5 μg of protein were separated by SDS-PAGE and transferred to a nylon membrane (Poly Screen PVDF; Fisher Scientific, Pittsburgh, PA). Polyclonal antibodies specific to human CLDN-7 (Zymed Laboratories, San Francisco, CA) were used according to the manufacturer's instructions to bind CLDN-7. The specificity of the CLDN-7 antibodies was tested by binding proteins isolated from CLDN-2 gene-transfected cells. No cross-binding was detected, although strong expression of CLDN-2 was noted when using CLDN-2-specific antibodies that were purchased from Zymed, indicating that those antibodies are highly specific.

## Antibody blockage of HIV infection

Polyclonal antibodies against gp120 or gp160 were obtained from the NIH HIV Reagents Program (Rockville, MD), and gp41-specific monoclonal antibody (mAB) was obtained from Virogen (Watertown, MA). Polyclonal antibodies against CLDN-7 were purchased from Zymed Laboratories (0.25 mg/ml) or made from rabbits using a peptide of extracellular domain 1 of CLDN-7 with the sequence CVTQSTGMMSCKMYD, from position 54 to 68 (concentration 0.85 mg/ml). The homology of the amino acid sequence of this peptide is 73% or less of he corresponding sequences of other human claudins. Antibodies were added into both viral stocks and cell cultures for 10 minutes prior to infection at room temperature. The final concentration of antibodies in the infection medium was a 1:100 dilution. Four hours post-infection, the cell cultures were washed once, and fresh culture medium added. Virus with no antibody (control) was also added and incubated at room temperature for 10 minutes prior to infection.

## RT-PCR to quantify mRNA levels of CLDN-7

We used the quanidinium thiocyanate method to isolate RNA. RNA isolated from approximately 10^5 ^cells was suspended in 20 μl of water. The isolated RNA was reverse transcribed, using AMV reverse transcriptase from Roche with oligo-dT as the primer. An aliquot of the cDNA was used for RT-PCR with primers 5'-CTCCTCTGACTTCAACAGCG and 5'-TGTTGCTGTAGCCAAATTCG to detect glyceraldehydes-3-phosphate dehydrogenase (GAPDH) cDNA. The primers of CLDN-7 have been previously described [26]. The expected sizes of the PCR products were 121 bp (GAPDH) and 252 bp (CLDN-7^211^).

## Competing interests

The author(s) declare that they have no competing interests.
